# Bayesian Cohort and Cross-Sectional Analyses of the PINCER Trial: A Pharmacist-Led Intervention to Reduce Medication Errors in Primary Care

**DOI:** 10.1371/journal.pone.0038306

**Published:** 2012-06-07

**Authors:** Karla Hemming, Peter J. Chilton, Richard J. Lilford, Anthony Avery, Aziz Sheikh

**Affiliations:** 1 School of Public Health, Epidemiology and Biostatistics, University of Birmingham, Birmingham, United Kingdom; 2 Division of Primary Care, University of Nottingham, Nottingham, United Kingdom; 3 eHealth Research Group, Centre for Population Health Sciences, The University of Edinburgh, Edinburgh, United Kingdom; Queen’s University Belfast, United Kingdom

## Abstract

**Background:**

Medication errors are an important source of potentially preventable morbidity and mortality. The PINCER study, a cluster randomised controlled trial, is one of the world’s first experimental studies aiming to reduce the risk of such medication related potential for harm in general practice. Bayesian analyses can improve the clinical interpretability of trial findings.

**Methods:**

Experts were asked to complete a questionnaire to elicit opinions of the likely effectiveness of the intervention for the key outcomes of interest - three important primary care medication errors. These were averaged to generate collective prior distributions, which were then combined with trial data to generate Bayesian posterior distributions. The trial data were analysed in two ways: firstly replicating the trial reported cohort analysis acknowledging pairing of observations, but excluding non-paired observations; and secondly as cross-sectional data, with no exclusions, but without acknowledgement of the pairing. Frequentist and Bayesian analyses were compared.

**Findings:**

Bayesian evaluations suggest that the intervention is able to reduce the likelihood of one of the medication errors by about 50 (estimated to be between 20% and 70%). However, for the other two main outcomes considered, the evidence that the intervention is able to reduce the likelihood of prescription errors is less conclusive.

**Conclusions:**

Clinicians are interested in what trial results mean to them, as opposed to what trial results suggest for future experiments. This analysis suggests that the PINCER intervention is strongly effective in reducing the likelihood of one of the important errors; not necessarily effective in reducing the other errors. Depending on the clinical importance of the respective errors, careful consideration should be given before implementation, and refinement targeted at the other errors may be something to consider.

## Introduction

### Frequentist and Bayesian Statistics: A Short Primer

The Bayesian perspective on statistical inference encapsulates an inductive philosophical approach to statistics [1], [2]. The Bayesian paradigm provides several important benefits. One of these benefits might be under-appreciated, given that many interpret frequentist, sometimes also known as classical, confidence intervals as providing intervals which contain the true effect with some particular probability, say a 95% probability, when in fact they provide information on likelihood of such an observation over the course of many repetitions of the trial (so long as models are well specified and sample sizes sufficiently large). However, the Bayesian approach actually provides intervals such that the posterior probability of the clinical effects lying within the interval is indeed the afore-mentioned probability, say 95%, and so therefore allows straight-forward interpretation of inferences on the clinical important concepts. Whilst Bayesian inferences do also allow direct statements about probabilities of effects, the Bayesian paradigm is also upheld for moving the focus of interpretation away from dichotomising whether the data suggest the intervention works or not, i.e. by not claiming a “significant” or “null result”, and more towards estimates of effect sizes, and so switching focus to how clinically important the intervention is [3].

Bayesian methods also allow for the incorporation of evidence additional to the conventional data [4], [5]. This additional evidence is called a prior distribution and summarises information from external sources. This external evidence might take the form of subjective opinions or down-weighted less rigorous, or indirectly related, results from previous studies [1], [6]. Opinion-based prior distributions might, for example, be based on sceptical opinions, perhaps reflecting the belief that the intervention might be less effective in routine practice (compared to the clinical trial setting); or optimistic opinions reflecting the belief that the intervention might be more beneficial in practice (compared to the clinical trial setting) perhaps due to more flexible dosing titration for example [7], [8].

The use of Bayesian statistics within the applied medical literature is growing, especially in the remits of early phase trials, data monitoring, and adaptive randomisation techniques [9], [10]; and towards the other end of the evidence hierarchy, Bayesian methods are increasingly being used within evidence synthesis [11], [12]. However, use of Bayesian methods within the evaluation of large-scale definitive trials is more limited [13]–[15]. In addition, some Bayesian applications, whilst allowing for very complex models, use off-the-shelf uninformative or vague prior distributions (a prior which places equal weight across the entire range of values and reflects no informative external information), and so don’t fully incorporate the Bayesian paradigm, which includes knowledge from all sources including expert opinions, [16], [17]. Others, where no other prior knowledge is available, use Bayesian methods to allow the data to dominate, and again use uninformative or vague prior distributions [18].

The Bayesian approach also naturally incorporates a sensitivity analysis, although it is quite rare to do this, considering the impact of various alternative prior distributions and sometimes alternative models too [13]. Whilst analysis consistent with that pre-specified in the trial protocol is essential to reduce selection biases, sensitivity analyses acknowledge that many findings are later rebutted [19].

Whilst frequentist methods can and do sometimes incorporate sensitivity analyses, and frequentist analyses reporting only confidence intervals also shift the focus away from statistical significance, it is only the Bayesian approach that provides a unified framework for this in addition to the incorporation of expert opinions and intuitive interpretation of confidence intervals.

### The PINCER Trial

Medication errors are common in general practice and are associated with a substantial burden of iatrogenic harm. Our previous systematic review of the literature found a dearth of evidence from high quality randomised controlled trials on how best to minimise this risk of patient harm [20]. Building on related theoretical, descriptive and qualitative work, we developed and piloted the PINCER intervention, which was then formally investigated [21]–[23].

In summary, PINCER (registration number ISRCTN21785299) was a large two arm UK-based cluster randomised controlled trial, with ethical approval for the trial from the Nottingham ethics committee (reference number: 05/Q2404/26). This additional post-hoc analysis did not require ethical approval as it constitutes a secondary re-analysis of existing data and elicitation of the views of university academics. The trial was undertaken in 72 practices that aimed to establish the effectiveness and cost-effectiveness of a complex pharmacist-led IT-enabled intervention compared with simple feedback to practices in reducing the rate of clinically important errors in medicines management in general practice. The three main outcomes of interest related to reducing the proportions of patients deemed at risk of the following potentially serious medication errors:

- Patients with a peptic ulcer who are prescribed a non-steroidal anti-inflammatory drug (NSAID);
- Patients with asthma who are prescribed a beta-blocker;
- Patients who are older than 75 years, prescribed an angiotensin converting enzyme (ACE) inhibitor or loop diuretic, and who have not had a renal or electrolyte assessment in the past 15 months.

Further details of the PINCER trial and its main findings are available from the trial protocol [24], and the published final reports, with key findings discussed below [25], [26].

### Bayesian Analysis of the PINCER Trial

In this paper, we report the first Bayesian analysis of a service delivery intervention [27]. These additional post-hoc Bayesian analyses would, we anticipated, allow incorporation of subjective opinions from experts in the field; intuitive interpretation of confidence intervals; and also provide a framework from within which it was possible to evaluate robustly sensitivity to model specifications. We envisage that this would assist policymakers and clinicians in making decisions on the basis of the current trial findings (rather than giving estimates of how effective the trial might prove if it was to be repeated). In the event, two different yet legitimate statistical approaches yielded statistically significant and null results for the same parameter of effectiveness; these results were compared with the reported trial analysis which was pre-specified in the trial protocol. This scenario enabled a contrast to be drawn between the way results might be presented to decision makers under frequentist and Bayesian analytical frameworks, which naturally incorporates sensitivity analyses.

## Methods

### Experts

We purposefully selected a multi-disciplinary panel of individuals with established interests/expertise in prescribing safety in primary care contexts, whether from a clinical and academic, or health policy context. The sampling frame was developed through discussions amongst the research team on expert contacts, who themselves had backgrounds in primary care, public health, epidemiology and statistics, and our familiarity with the research literature. The experts had no role in designing the study or vested interest in its outcome. As one of the world’s first randomised controlled trials aiming to improve prescribing safety in a primary care context, the PINCER trial design is well known in specialist circles. Furthermore, the detailed trial protocol was published and available [24]. It was thus very likely that the experts had a good appreciation of the trial’s aims and methods, which would inform their estimates of likely effectiveness. We provided members of our expert panel with a short summary of the trial, prior to their completing the questionnaire, in an attempt to mitigate any differences in prior knowledge about the study.

### Elicitation Methods

Expert opinions were elicited using a structured interview form, and followed best practice recommendations [4], [5]. This form was emailed electronically to all experts. Experts had the option of completing the elicitation form themselves or through a structured telephone interview with members of the study research team (KH or PC). All opinions were elicited before any of the trial results were presented at conferences, or published in peer-reviewed or other publications. Please see Appendix S1 for a copy of the elicitation form.

The elicitation form included information on the three main outcomes, including the estimated baseline event rate. Experts were then asked for their opinion on the direction of any change they expected in rates of errors, for both the intervention and control groups. This very basic information was elicited to allow validation of the consistency of the information elicited in the more detailed information.

Experts were then informed that the trial was designed to detect a relative reduction of 10% in the control arm and 50% in the intervention arm. Provided with this information experts were asked to provide their “best guess” of the relative reduction (−0% to −100% for a decreasing rate, or +0% to +100% for an increasing rate) in both arms; along with maximum and minimum plausible ranges, which we described as representing their 95% confidence interval. For example, the rate of Potential Error 1 was estimated to be about 6% before the trial. An expert believing that this might reduce to 3% in the control arm would need to express this as a −50% relative reduction; and a similar opinion would need to be provided for the change in the intervention arm. Experts were told that their plausible ranges should represent an effect size at the extreme 2·5% and 97·5% limits to roughly represent their 95% confidence intervals.

### Summarising Beliefs Elicited

The beliefs elicited from each of the experts were pooled to obtain a collective prior distribution. Two different interpretations of the elicited beliefs were considered.

The first interpretation of the beliefs was an optimistic interpretation. Here it was assumed that the elicited maximum (or minimum) plausible range in the intervention group was concordant with the elicited maximum (or minimum) plausible range in the control group. For example, suppose an expert specified a reduction in the intervention arm of 50%, with 95% interval 40% to 60%; and a reduction in the control arm of 20%, with 95% interval 10% to 30%. This expert therefore believed that the average relative risk for the intervention was 2.5 (i.e. 50/20). Assuming concordance, between intervention and control arms, of maximum and minimum values would mean that if in the intervention arm the reduction was the at the lower of the specified value (i.e. 40%), then in the control arm the reduction would also be at the lower end of the specified value (i.e. 10%). Collectively a reduction of 40% in the intervention arm and 10% in the intervention arm equates to a relative risk of 4 (i.e. 40/10). Similarly, assuming concordance of the upper beliefs specified in both the intervention (60% reduction) and control arms (30% reduction) would equate to a relative risk of 2 (i.e. 60/30). So under this interpretation the belief elicited for the relative risk has a 95% interval between 2 and 4.

The second interpretation was conservative. It might be the case that the expert in the example above did not mean to specify the maximum and minimum plausible ranges in a concordant way. So, in the example above, a non-concordant interpretation of a 95% range 40% to 60% in the intervention arm and 10% to 30% in the control arm, gives a maximum relative risk of 6 (i.e 60/10) and a minimum of 1·3 (i.e. 40/30). This second interpretation not assuming concordance between maximum (or minimum) intervention and control limits, results in confidence ranges which are wider and so less certain, and so is called a conservative interpretation.

Both the conservative and optimistic beliefs for the relative risks were then pooled over all experts, assuming normality on the log scale, by taking averages of means and standard deviations, obtained from the upper and lower confidence ranges, of individual opinions. This then formed two (conservative and optimistic) subjective prior distributions for the odds of reduction in error rate comparing intervention to control.

### Models for the PINCER Trial Data

The original analysis of the trial data was from a frequentist perspective [26]. For the three primary outcomes, the original trial analysis reported odds of reduction (intervention versus control) at six months, estimated using random effects logistic models with clustering at the practice level and adjustment for baseline presence of the error and several practice level variables, with 95% confidence intervals. Practice level variables used within the adjustments were: Index of Multiple Deprivation (IMD) score; list size (three strata <2500, 2500–6000, and >6000 patients); centre (Manchester and Nottingham); and training status of the practice (Yes/No) [24], [25]. Only individuals with data at baseline and six months were included in the original trial analysis. This trial-reported analysis is consistent with recommendations of how to analyse paired (i.e. baseline and follow-up) data, using what is often described as an ANCOVA approach, which consists of a regression type analysis including the baseline value as a covariate [28]. This is henceforth referred to as the trial reported analysis in all reporting of results and tabulations below.

In cluster randomised controlled trials, the study population often forms a cohort population who are followed-up, and baseline and follow-up measurements obtained on the same individuals. Alternative designs, known as cross-sectional, use different individuals at before and after time points [29]. The PINCER study includes a mixture of the cohort and cross-sectional design: some individuals are included at both baseline and follow-up, whereas others are not.

Where there is a positive correlation between measurements, then it has been established that an analysis that does not acknowledge pairing is conservative; and that more precise confidence intervals will be obtained from the analysis which acknowledges the pairing [30]. However, a paired analysis necessitates the exclusion of observations with either the baseline or follow-up measurement missing. Excluding observations from an analysis can have two consequences. The first of which is a possible loss of precision by excluding observations, the size of the dataset is reduced. The second consequence is the possibility of biased estimates since those observations excluded from the analysis might not be representative. There are techniques available to mitigate the loss of information from observations which have some missing covariate data (e.g. multiple imputation), but even in a Bayesian framework these necessitate an assumption of the missing data being missing at random.

So, an alternative way of analysing the PINCER study is to consider the study as two cross-sectional studies, one before and one after the intervention. This might or might not bring benefits in terms of precision (depending on whether the benefit from the pairing is greater than the benefit from the absolute increase in numbers of observations). However, by not having to exclude any observations, the cross-sectional analysis will not make any assumptions about the nature of the reasons for missing follow-up or baseline information, and so should provide the least biased estimates.

We therefore compared two models, a cohort type approach (i.e. consistent with the trial reported analysis) and cross-sectional approach. In the cohort approach only individuals with both before and follow-up measurements were included, the outcome modelled was the presence of error at follow-up and covariates included an indicator of intervention arm, and an indicator for whether that individual was being prescribed in error at baseline. In the cross-sectional analyses, the outcome was presence of error and covariates included in the model were an indicator of time; an indicator of intervention or control group; and an interaction between time and intervention. The interaction term summarises the effect of treatment after adjustment for baseline differences. All models used logistic regression, adjusted for GP practice level categorical variables (IMD score, list size and training status of practice) and included a random effect for GP practice.

In summary, re-analysed the PINCER data from a frequentist perspective but using a cross-sectional approach, this analysis is referred to as the frequentist cross-sectional analysis. This was compared to the trial reported analysis which we call the frequentist cohort-analysis. The frequentist models, including only the PINCER trial data, were fitted using StataSE version 11.2. We then additionally combined the cohort and cross-sectional analyses with the elicited prior distributions (methods outlined below).

### Methods for Combining Prior Distributions with Trial Data

For the Bayesian analysis we generated Bayesian posterior distributions combining the elicited prior distributions with the PINCER trial data, analysed from both a cohort and cross-sectional perspective. All Bayesian analyses were performed using the WinBUGS software with 100,000 iterations after allowing for a 20,000 iteration burn-in and checking for convergence using several common measures [31]. Summary estimates provided are median and 95% Credible Intervals (CrI), which can be interpreted as one would like to interpret a frequentist confidence interval (i.e. an interval which contains the effect with 95% probability). For parameters other than the treatment effect, we used standard uninformative prior distributions [32]: uniform with range 0 and 100 for the standard deviations; and normal distribution centred at 0 and with variance 1,000 for other parameters. Finally, we compared the analysis using the elicited optimistic and conservative prior distributions with that of an uninformative prior distribution for all parameters (i.e. by also including a vague prior for the treatment effect parameter normally distributed with mean 0 and variance 1,000). These thus formed the Bayesian cohort and Bayesian cross-sectional analyses.

## Results

**Figure 1:**
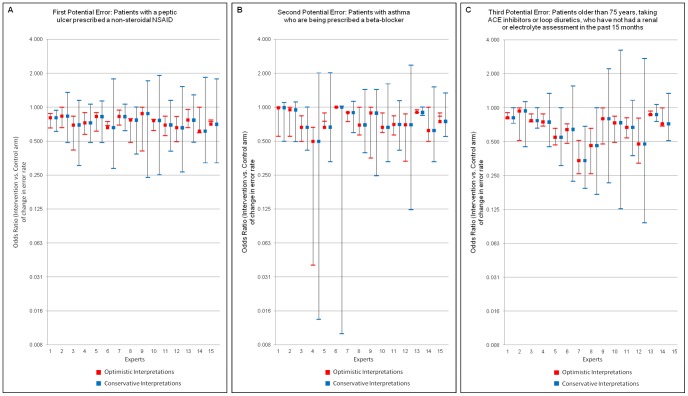
Elicited expert opinions on three outcomes, under conservative and optimistic interpretations.

**Figure 2:**
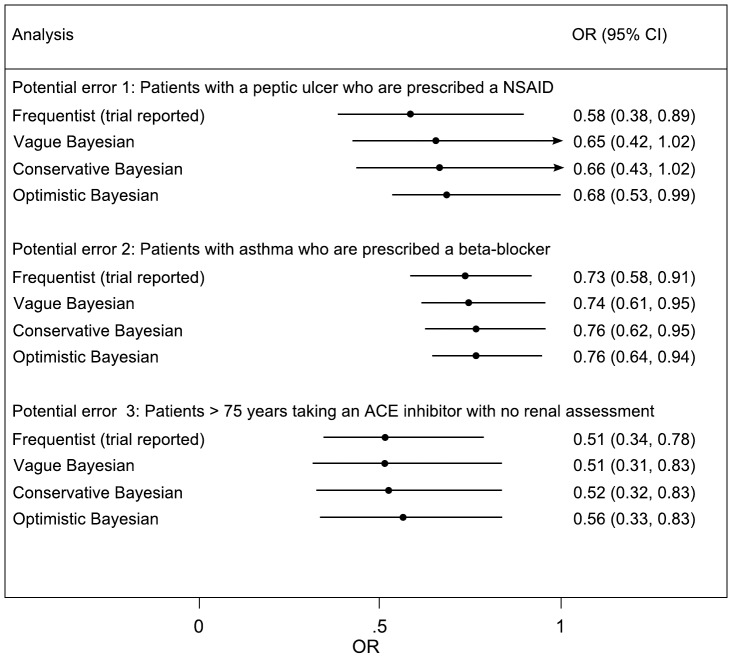
Cohort analysis of odds of error in control to intervention arm in the PINCER trial. The conservative and optimistic priors are elicited expert priors. CI refers to frequentist confidence interval or credible interval for Bayesian analyses.

**Figure 3:**
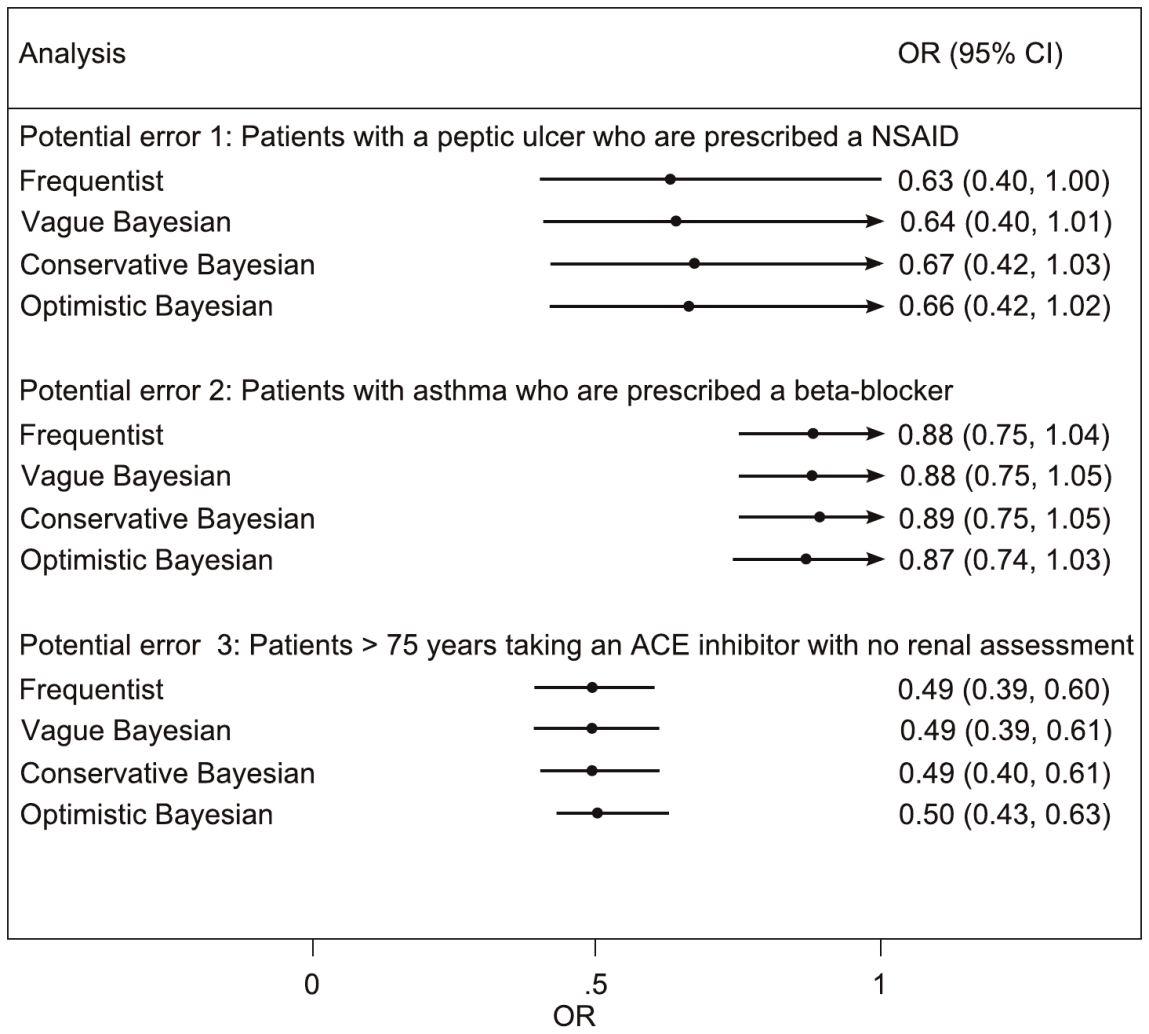
Cross-sectional analysis of odds of error in control to intervention arm in the PINCER trial. The conservative and optimistic priors are elicited expert priors. CI refers to frequentist confidence interval or credible interval for Bayesian analyses.

Thirty-four experts were approached via email. Of these, 15 agreed to participate; a further four responded stating they would not like to participate (two because they had already seen the trial results; one forwarded to a colleague; and one who was too busy); and the remaining 15 failed to respond. Of the 15 responses obtained, 11 responded directly by electronic completion of the form and four responses were obtained by telephone interview. One expert, whilst providing opinions for the first two outcomes, did not feel able to provide an opinion on the third outcome. Opinions provided by each of the experts are presented in Figure 1. The conservative interpretations of the elicited opinions are much less precise than those of the optimistic interpretation. Pooling over experts the conservative and optimistic prior distributions are shown in Table 1. Results are presented from the frequentist analysis for the three outcomes and for both the cohort (Figure 2) and cross-sectional analyses (Figure 3).

**Table 1:** Elicited priors for log odds ratios and corresponding 95% Credible Intervals for the odds ratios.

Outcome		OptimisticPrior	Conservative Prior
**Potential** **Error 1**	Log OR (mean, var)	N(−0.30,0.35)	N(−0.30,0.63)
	OR [LCrI, UCrI]	0.74 [0.59,0.95]	0.74 [0.34,1.63]
**Potential** **Error 2**	Log OR (mean, var)	N(−0.28,0.46)	N(−0.28,0.76)
	OR [LCrI, UCrI]	0.76 [0.50,1.15]	0.76 [0.24, 2.34]
**Potential** **Error 3**	Log OR (mean, var)	N(−0.42,0.36)	N(−0.42,0.62)
	OR [LCrI, UCrI]	0.66 [0.51,0.85]	0.66 [0.31,1.39]

### Reducing the Proportion of Patients with a Peptic Ulcer who are Taking a NSAID

Experts generally agreed that the intervention should reduce these rates to a greater degree in the intervention compared to the control arm. The average estimated reduction (relative to baseline) was 40% (95%CrI 15, 60) in the intervention, and 17% (95%CrI 4, 34) in the control arm. This translated into a collective averaged belief (i.e. prior) of odds of reduction of error in intervention practices of 0·74 (95%CrI 0·34, 1·63) under a conservative interpretation; and a prior odds of reduction of 0·74 (95%CrI 0·59, 0·95) under an optimistic interpretation. Both these prior distributions are centred on effect estimates which are slightly smaller than that estimated by the trial reported analysis and with less certainty (OR in trial reported analysis: 0·58 (95%CI 0·38, 0·89)).

The trial reported analysis for this variable included 3,434 paired observations; whereas the cross-sectional analysis included 7,664 individual observations (translating to an exclusion of 796 observations in the cohort analysis). Under the frequentist and Bayesian approaches, both the cohort and cross-sectional analyses were reasonably similar (Figures 2 and 3). This is suggestive of little difference in both bias and precision between the two methods, although the cohort analysis is somewhat more precise.

### Reducing the Proportion of Patients with Asthma who are Prescribed a Beta-blocker

Experts generally agreed again that the intervention should reduce these rates to a greater degree in the intervention compared to the control arm. The averaged best guess relative (to baseline) reduction was 37% (95%CrI 14, 64) in the intervention and 20% (95%CrI 6, 39) in the control arm. This translated into a prior odds of reduction of 0·76 (95%CrI 0·24, 2.34) under a conservative interpretation; and a prior odds of reduction of 0·76 (95%CrI 0·50, 1.15) under an optimistic interpretation. Again, both these prior distributions were centred on effect estimates which were slightly smaller than that estimated by the trial’s reported analysis, with the optimistic prior mirroring closely the trial reported analysis (OR 0·73; 95%CI 0·58, 0·91).

The trial reported analysis for this variable included 39,235 paired observations; whereas the cross-sectional analysis included 82,076 observations: meaning that the trial reported analysis excluded 3,606 observations. Under the cross-sectional analysis, both the frequentist and Bayesian analysis suggested that the magnitude of the intervention may be less than that suggested by the trial reported analysis, for example under the Bayesian analysis with vague prior OR 0·88 (95%CI 0·75, 1·05). These translate into estimates of reduction in odds of error of 27% and 12%. This difference in findings between these two approaches suggests that whilst power may be increased from the pairing (hence tighter confidence and credible intervals under the cohort analysis) patients without both before and after measurements are less likely to have experienced a benefit from the treatment (Figures 2 and 3).

### Reducing the Proportion of Patients Who are Older than 75 Years Taking an ACE Inhibitor or Loop Diuretic, Who have not had A Renal or Electrolyte Assessment in the Past 15 Months

Experts generally agreed once again that the intervention should reduce these rates to a greater degree in the intervention compared to the control arm. The average best guess relative (to baseline) reduction was 43% (95%CrI 20, 61) in the intervention, and 20% (95%CrI 7, 35) in the control arm. This translated into a prior odds of reduction of 0·66 (95%CrI 0·31, 1·39) under the conservative interpretation; and a prior odds of reduction of 0·66 (95%CrI 0·51, 0·85) under an optimistic interpretation. These prior distributions were broadly similar to that estimated by the trial’s reported analysis (0·51; 95%CI 0·34, 0·78).

The trial reported analysis for this variable included 8,185 paired observations; whereas the cross-sectional analysis included 19,251 observations: meaning that the trial reported analysis excluded 2,881 observations. Under the cross-sectional analysis, both the frequentist and Bayesian analysis, suggested the magnitude of the intervention was very similar to that suggested by the trial reported analysis, but that the precision was increased under the cross-sectional analysis (Figures 2 and 3).

## Discussion

Our Bayesian re-analysis of the PINCER trial, eliciting opinions from experts in the field and incorporating these into a Bayesian sensitivity analysis, additionally considering the influence of modelling the data as cross-sectional or cohort, demonstrate that the data consistently suggest that the intervention reduces the likelihood of patients (>75years) on long term ACE inhibitor medication, not having relevant renal checks, by on average somewhere in the region of a 50% relative reduction, and that this reduction would almost certainly be between 20% and 70%. However, for the other two main outcomes, prescription of NSAIDs in patients with a peptic ulcer and patients with asthma who are prescribed a beta-blocker the evidence for effectiveness is less certain and effect sizes probably smaller.

Policymakers and clinicians need to make decisions on the basis of current trial findings. Reliance on P-values dichotomises results and may mean that conclusions toggle from ‘non-significant’ to ‘significant’ depending on the analysis method. Confidence intervals are beneficial in this regard, but frequentist confidence intervals are prone to miss-interpretation and so Bayesian analyses can be useful here. It might be argued that frequentist confidence intervals are often good approximations to Bayesian credible intervals, but with today’s computational power it is not necessary to make such approximations. Furthermore, a Bayesian approach, by considering the influence of choice of prior distribution on inferences, naturally allows the incorporation of sensitivity analyses. Sensitivity analyses are readily incorporated into a frequentist analysis, but where sensitivity to analysis choice is shown, then the impact of the sensitivity analysis can be at odds with the desire to follow analysis methods pre-specified as primary in the trial protocol.

Perhaps surprisingly, given the Bayesian approach might be thought of as increasing the effective sample size, it has previously been shown that Bayesian analyses often results in estimates which are less certain when compared to their frequentist counterparts [12]. This can be because the Bayesian model correctly acknowledges that all model parameters are estimated with uncertainty, whereas in a frequentist approach some parameters, such as variance parameters in random effects models, are assumed to be estimated without uncertainty [12], [13]. Methods which do not acknowledge all parameter uncertainty will inappropriately attenuate the estimated uncertainty.

The collective expert prior distributions, for all of the three outcomes, were generally wider and less optimistic compared to the frequentist estimates from the trial data. The experts were therefore cautious in their certainty, but were optimistic for the intervention. The three collective priors distributions, for each of the three outcomes, were very similar suggesting that the experts could discriminate little between the potential for effectiveness of the intervention on each of the outcomes. Because of the very large study sample sizes, their prior opinions did not influence the posterior to a great extent. When results toggle from ‘non-significant’ to ‘significant’ according to the analysis undertaken, the Bayesian approach provides a guide for decision makers by steering away from the frequentist P-value, focuses on the range of uncertainty, and does not dichotomise the results into yes, the intervention is effective, or no, the intervention is not effective.

In addition to considering influence of expert opinions, we also considered sensitivity to model choice. It is widely recognised that the most efficient way to analyse paired before and after data are by using ANCOVA methods [28]. This is consistent with viewing a longitudinal study, like PINCER, as a cohort study, and indeed this analysis was pre-specified in the trial protocol. Whilst this recommendation is not disputed, in practical applications it is important to consider the implications of excluding non-paired observations on both precision and bias. An alternative approach to analysis is thus to consider the study as a series of cross-sectional studies, which, whilst not acknowledging the pairing of observations, does not require observations without both before and after measurements to be excluded.

### Strengths and Limitations of this Work

To our knowledge this is the first trial of a service level intervention that has undertaken both frequentist analyses and also reported on parallel Bayesian analysis, with subjective (as opposed to uninformative) prior distributions. We are also we believe the first to report the analysis of a cluster trial as a mixture of the cohort and cross-sectional design. We took care to ensure that members of our expert panel were not aware of the trial results prior to eliciting their expert estimates, although with hindsight this might not have been necessary [1]. Furthermore, to mitigate any differences in knowledge about the study with provide all experts with basic information on the values PINCER was powered to detect. This may have inadvertently acted as an anchor, but elicited values did not give cause for concern. The similarity between the opinions of the experts and the trial data gives added confidence in the external validity of the findings from this Bayesian analysis.

This work is however not without its potential limitations. Opinions were elicited on the relative effects of the intervention compared to the baseline for the control and intervention arms independently, as recommended by O’Hagan and Stevens [2]. An alternative method would be to elicit opinions for the relative effects of the control to intervention arms. However, because the outcome of interest was the odds ratio this would have meant eliciting a prior on an odds ratio scale, which may have been confusing to the experts. Another potential limitation is that we did not clearly specify the follow-up time for primary outcomes (six months) when eliciting opinions, but as noted above, given experts overall likely familiarity with the trial design, this is unlikely to have had a major impact on the findings from this work.

### Implications for Policy, Practice and Future Research

The trial reported analysis should be dictated by the protocol and so with this in mind the PINCER trial should be analysed as a cohort study as originally intended. The trade-off in precision between the two methods considered (cross-sectional versus cohort) depends on the loss to follow-up. Estimates of these rates of losses to follow-up could conceivably be estimated prior to the start of a study and so required sample sizes (adjusted for loss to follow-up) could be compared between both the cohort approach and the cross-sectional approach. However, precision is not the only important consideration and implications for bias are important too. At the very extreme, analysis by cohort, excluding all cases without before and after measurements, could indivertibly exclude all patients who subsequently died of a medication error, which is clearly not a fair comparison.

Although this additional analysis has, at face value, generated results that are similar to the findings of those derived from the trial reported analysis, it has also succeeded in presenting the results as degrees of belief, the axiomatic method for formal decision models, including health economic models. In this particular study it illustrates a putative advantage from Bayesian methods when different approaches to data analysis, neither of which can be said to be wrong, proved a scenario in which results are either significant or null according to method used. Finally, the Bayesian approach of eliciting expert opinion prior to the commencement of the study allows proper appreciation of equipoise and might help determine whether a trial is ethical [1]. Bayesian analyses of data should therefore be encouraged and pursued more commonly.

This complex and post-hoc analyses of the PINCER trial confirm that the intervention is likely to be effective in reducing the potential for medication harm in general practice, but that the only outcome for which the data suggest an unequivocal reduction in risk is failure to carry out renal checks in those (>75 years) on long term ACE inhibitor medication. For the other two outcomes, prescription of beta-blockers in patients with asthma, and NSAIDs in patients with peptic ulcers, although the evidence points towards a reduction in risk, this evidence is less certain.

## Supporting Information

Appendix S1 **Elicitation Form.**
(DOCX)
