# Supplementary material for: Bayesian Cohort and Cross-Sectional Analyses of the PINCER Trial: A Pharmacist-Led Intervention to Reduce Medication Errors in Primary Care
Source: PLoS One. 2012 Jun 7;7(6):e38306. doi: 10.1371/journal.pone.0038306 (PMC3369915; doi:10.1371/journal.pone.0038306)
Supplement: Appendix S1 — Elicitation Form. (DOCX) [file pone.0038306.s001.docx]

**Appendix 1: Elicitation Form**

**Bayesian Elicitation for the effectiveness of a pharmacist led IT intervention to reduce medication errors (The PINCER trial)**

- What is the PINCER trial?

The PINCER trial (*www.pincertrial.org*) is a pragmatic cluster randomised trial to determine the effectiveness of a pharmacist led IT complex intervention in reducing the proportion of patients at risk from potentially serious medication errors. Each of 72 GP practices has been randomly assigned to either the intervention arm, or a control arm. In both arms of the trial, a purpose built software package is used to identify patients who are at risk of medication error, by identifying those patients who:

1. Have a history of a peptic ulcer, but who are currently being prescribed a non-selective non-steroidal anti-inflammatory drug (NSAID).
2. Have a diagnosis of asthma, but who are being prescribed beta blockers.
3. Are 75 years or older and are being prescribed angiotensin converting enzyme (ACE) inhibitors or loop diuretics, but without an assessment of renal function or electrolytes in the previous 15 months.

In the *control arm* lists of all such identified patients are simply given to the practices. In the *intervention arm*, a pharmacist discusses these outcomes with the practice team, to identify ways in which such errors could be avoided in the future, and all such identified patients are invited for an individual consultation during which their medications are reviewed.

The main outcomes of the study are the proportion of patients falling into each of the categories 1 to 3 above, called potential errors:

**Potential error 1:** Proportion of patients with a history of a peptic ulcer, who are being prescribed a non-selective NSAID (estimated from a pilot study to be about 6% before the intervention).

**Potential error 2:** Proportion of patients with asthma, who are being prescribed beta-blockers (estimated from a pilot study to be about 2% before the intervention).

**Potential error 3:** Proportion of patients older than 75 years taking ACE inhibitors or loop diuretics, who have not had a renal or electrolytes assessment within the past 15 months (estimated from a pilot study to be about 18% before the intervention).

- What might the effectiveness of this complex intervention be?

It is expected that at the end of the study the proportion of patients falling into each of the three error groups will be reduced in both the control and intervention groups. It is hoped that the reduction in potential errors will be greatest in the intervention group. The trial was powered to detect a difference of 10% in the control arm and 50% in the intervention arm (for example it is hoped that the intervention will reduce the proportion of patients falling in the potential error 3 group from 18% to 9%).

- We are attempting to elicit clinical expert opinions on how effective this intervention is thought to be and would be very grateful if you could complete the following short questionnaire.
- What is your opinion of the likely impact this complex intervention will have on the first potential error: *reducing the proportion of patients with a peptic ulcer who are being prescribed a non-steroidal NSAID (estimated to be about 6%)*?

1. ***Do you think it is likely that the proportion of patients who fall into this potential for medication error group will be reduced?***

| *Control Arm* | *Yes* | *No* | *Don’t know* |
| --- | --- | --- | --- |
| *Intervention Arm* | *Yes* | *No* | *Don’t know* |

1. ***The trial was powered to detect a reduction in the proportion of patients experiencing this potential error of 10% in the control arm and 50% in the intervention arm. What do you think these reductions might be?***

|  | **Minimum Plausible Percentage Change^[[1]](#footnote-1)^** | **Your Best Guess** | **Maximum Plausible Percentage Change^1^** |
| --- | --- | --- | --- |
| Control Arm | %   in error rate | %   in error rate | %   in error rate |
| Intervention Arm | %   in error rate | %   in error rate | %   in error rate |

1. ***Please try to represent your opinion on the scale below by distributing a total of 100 points between the lines to reflect your subjective beliefs about each percentage change occurring.***

*For example,* Total = 100

|  |  | |  | |  | |  | |  | | 10 | | 10 | | **15** | | 15 | | 15 | | 15 | | 10 | | 10 | |  |
| --- | --- | --- | --- | --- | --- | --- | --- | --- | --- | --- | --- | --- | --- | --- | --- | --- | --- | --- | --- | --- | --- | --- | --- | --- | --- | --- | --- |
|  | | -60 | | -55 | | -50 | | -45 | | -40 | | -35 | | **-30** | | **-25** | | -20 | | -15 | | -10 | | -5 | | **0** | |

*This would mean you feel there is a* ***15%*** *chance that the percentage reduction would be between* ***30****% and* ***25****%, and that there would be no chance that the intervention would increase the error rate.*

For the control arm:

|  | |  | |  | |  | |  | |  | |  | |  | |  | |  | |  | |  | |  | |  | |  | |  | |  | |  | |  | |  | |  | |  | |
| --- | --- | --- | --- | --- | --- | --- | --- | --- | --- | --- | --- | --- | --- | --- | --- | --- | --- | --- | --- | --- | --- | --- | --- | --- | --- | --- | --- | --- | --- | --- | --- | --- | --- | --- | --- | --- | --- | --- | --- | --- | --- | --- | --- |
|  | -80 | | -75 | | -70 | | -65 | | -60 | | -55 | | -50 | | -45 | | -40 | | -35 | | -30 | | -25 | | -20 | | -15 | | -10 | | -5 | | **0** | | +5 | | +10 | | +15 | | +20 | |  |
| **Control Arm**: Decreasing Error Rate (%) | | | | | | | | | | | | | | | | | | | | | | | | | | | | | | | | | | **Control Arm**:  Increasing Error Rate (%) | | | | | | | | | |

For the intervention arm:

|  | |  | |  | |  | |  | |  | |  | |  | |  | |  | |  | |  | |  | |  | |  | |  | |  | |  | |  | |  | |  | |  | |
| --- | --- | --- | --- | --- | --- | --- | --- | --- | --- | --- | --- | --- | --- | --- | --- | --- | --- | --- | --- | --- | --- | --- | --- | --- | --- | --- | --- | --- | --- | --- | --- | --- | --- | --- | --- | --- | --- | --- | --- | --- | --- | --- | --- |
|  | -80 | | -75 | | -70 | | -65 | | -60 | | -55 | | -50 | | -45 | | -40 | | -35 | | -30 | | -25 | | -20 | | -15 | | -10 | | -5 | | **0** | | +5 | | +10 | | +15 | | +20 | |  |
| **Intervention Arm**: Decreasing Error Rate (%) | | | | | | | | | | | | | | | | | | | | | | | | | | | | | | | | | | **Intervention Arm**:  Increasing Error Rate (%) | | | | | | | | | |

- What is your opinion of the likely impact this complex intervention will have on the second potential error: *reducing the proportion of patients with asthma who are being prescribed a beta-blocker (estimated to be about 2%)*?

1. ***Do you think it is likely that the proportion of patients who fall into this potential for medication error group will be reduced?***

| *Control Arm* | *Yes* | *No* | *Don’t know* |
| --- | --- | --- | --- |
| *Intervention Arm* | *Yes* | *No* | *Don’t know* |

1. ***The trial was powered to detect a reduction in the proportion of patients experiencing this potential error of 10% in the control arm and 50% in the intervention arm. What do you think these reductions might be?***

|  | **Minimum Plausible Percentage Change^[[2]](#footnote-2)^1** | **Your Best Guess** | **Maximum Plausible Percentage Change^1^** |
| --- | --- | --- | --- |
| Control Arm | %   in error rate | %   in error rate | %   in error rate |
| Intervention Arm | %   in error rate | %   in error rate | %   in error rate |

1. ***Please try to represent your opinion on the scale below by distributing a total of 100 points between the lines to reflect your subjective beliefs about each percentage change occurring.***

*For example,* Total = 100

|  |  | |  | |  | |  | |  | | 10 | | 10 | | **15** | | 15 | | 15 | | 15 | | 10 | | 10 | |  |
| --- | --- | --- | --- | --- | --- | --- | --- | --- | --- | --- | --- | --- | --- | --- | --- | --- | --- | --- | --- | --- | --- | --- | --- | --- | --- | --- | --- |
|  | | -60 | | -55 | | -50 | | -45 | | -40 | | -35 | | **-30** | | **-25** | | -20 | | -15 | | -10 | | -5 | | **0** | |

*This would mean you feel there is a* ***15%*** *chance that the percentage reduction would be between* ***30****% and* ***25****%, and that there would be no chance that the intervention would increase the error rate.*

For the control arm:

|  | |  | |  | |  | |  | |  | |  | |  | |  | |  | |  | |  | |  | |  | |  | |  | |  | |  | |  | |  | |  | |  | |
| --- | --- | --- | --- | --- | --- | --- | --- | --- | --- | --- | --- | --- | --- | --- | --- | --- | --- | --- | --- | --- | --- | --- | --- | --- | --- | --- | --- | --- | --- | --- | --- | --- | --- | --- | --- | --- | --- | --- | --- | --- | --- | --- | --- |
|  | -80 | | -75 | | -70 | | -65 | | -60 | | -55 | | -50 | | -45 | | -40 | | -35 | | -30 | | -25 | | -20 | | -15 | | -10 | | -5 | | **0** | | +5 | | +10 | | +15 | | +20 | |  |
| **Control Arm**: Decreasing Error Rate (%) | | | | | | | | | | | | | | | | | | | | | | | | | | | | | | | | | | **Control Arm**:  Increasing Error Rate (%) | | | | | | | | | |

For the intervention arm:

|  | |  | |  | |  | |  | |  | |  | |  | |  | |  | |  | |  | |  | |  | |  | |  | |  | |  | |  | |  | |  | |  | |
| --- | --- | --- | --- | --- | --- | --- | --- | --- | --- | --- | --- | --- | --- | --- | --- | --- | --- | --- | --- | --- | --- | --- | --- | --- | --- | --- | --- | --- | --- | --- | --- | --- | --- | --- | --- | --- | --- | --- | --- | --- | --- | --- | --- |
|  | -80 | | -75 | | -70 | | -65 | | -60 | | -55 | | -50 | | -45 | | -40 | | -35 | | -30 | | -25 | | -20 | | -15 | | -10 | | -5 | | **0** | | +5 | | +10 | | +15 | | +20 | |  |
| **Intervention Arm**: Decreasing Error Rate (%) | | | | | | | | | | | | | | | | | | | | | | | | | | | | | | | | | | **Intervention Arm**:  Increasing Error Rate (%) | | | | | | | | | |

- What is your opinion of the likely impact this complex intervention will have on the third potential error: *reducing the proportion of patients older than 75 years taking angiotensin converting enzyme inhibitors or loop diuretics, who have not had a renal or electrolyte assessment in the past 15 months (estimated to be about 18%)*?

1. ***Do you think it is likely that the proportion of patients who fall into this potential for medication error group will be reduced?***

| *Control Arm* | *Yes* | *No* | *Don’t know* |
| --- | --- | --- | --- |
| *Intervention Arm* | *Yes* | *No* | *Don’t know* |

1. ***The trial was powered to detect a reduction in the proportion of patients experiencing this potential error of 10% in the control arm and 50% in the intervention arm. What do you think these reductions might be?***

|  | **Minimum Plausible Percentage Change^[[3]](#footnote-3)^1** | **Your Best Guess** | **Maximum Plausible Percentage Change^1^** |
| --- | --- | --- | --- |
| Control Arm | %   in error rate | %   in error rate | %   in error rate |
| Intervention Arm | %   in error rate | %   in error rate | %   in error rate |

1. ***Please try to represent your opinion on the scale below by distributing a total of 100 points between the lines to reflect your subjective beliefs about each percentage change occurring.***

*For example,* Total = 100

|  |  | |  | |  | |  | |  | | 10 | | 10 | | **15** | | 15 | | 15 | | 15 | | 10 | | 10 | |  |
| --- | --- | --- | --- | --- | --- | --- | --- | --- | --- | --- | --- | --- | --- | --- | --- | --- | --- | --- | --- | --- | --- | --- | --- | --- | --- | --- | --- |
|  | | -60 | | -55 | | -50 | | -45 | | -40 | | -35 | | **-30** | | **-25** | | -20 | | -15 | | -10 | | -5 | | **0** | |

*This would mean you feel there is a* ***15%*** *chance that the percentage reduction would be between* ***30****% and* ***25****%, and that there would be no chance that the intervention would increase the error rate.*

For the control arm:

|  | |  | |  | |  | |  | |  | |  | |  | |  | |  | |  | |  | |  | |  | |  | |  | |  | |  | |  | |  | |  | |  | |
| --- | --- | --- | --- | --- | --- | --- | --- | --- | --- | --- | --- | --- | --- | --- | --- | --- | --- | --- | --- | --- | --- | --- | --- | --- | --- | --- | --- | --- | --- | --- | --- | --- | --- | --- | --- | --- | --- | --- | --- | --- | --- | --- | --- |
|  | -80 | | -75 | | -70 | | -65 | | -60 | | -55 | | -50 | | -45 | | -40 | | -35 | | -30 | | -25 | | -20 | | -15 | | -10 | | -5 | | **0** | | +5 | | +10 | | +15 | | +20 | |  |
| **Control Arm**: Decreasing Error Rate (%) | | | | | | | | | | | | | | | | | | | | | | | | | | | | | | | | | | **Control Arm**:  Increasing Error Rate (%) | | | | | | | | | |

For the intervention arm:

|  | |  | |  | |  | |  | |  | |  | |  | |  | |  | |  | |  | |  | |  | |  | |  | |  | |  | |  | |  | |  | |  | |
| --- | --- | --- | --- | --- | --- | --- | --- | --- | --- | --- | --- | --- | --- | --- | --- | --- | --- | --- | --- | --- | --- | --- | --- | --- | --- | --- | --- | --- | --- | --- | --- | --- | --- | --- | --- | --- | --- | --- | --- | --- | --- | --- | --- |
|  | -80 | | -75 | | -70 | | -65 | | -60 | | -55 | | -50 | | -45 | | -40 | | -35 | | -30 | | -25 | | -20 | | -15 | | -10 | | -5 | | **0** | | +5 | | +10 | | +15 | | +20 | |  |
| **Intervention Arm**: Decreasing Error Rate (%) | | | | | | | | | | | | | | | | | | | | | | | | | | | | | | | | | | **Intervention Arm**:  Increasing Error Rate (%) | | | | | | | | | |

- We would be grateful if you could provide the following information:

| **Your Name** |  |
| --- | --- |
| **Organisation** |  |
| **Email** |  |
| **Telephone** |  |
| **Years since graduation** |  |
| **Discipline** |  |
| **Title / position** |  |

THANK YOU VERY MUCH FOR YOUR TIME!

If you could please return your completed questionnaire to

k.hemming@bham.ac.uk

+

p.j.chilton@bham.ac.uk

| **The PINCER trial**  **--Contact us--** | | | |
| --- | --- | --- | --- |
| **Aziz Sheikh** | Centre for Population Health Sciences,  University of Edinburgh | **E:** | **aziz.sheikh@ed.ac.uk** |
| **Richard Lilford** | Public Health, Epidemiology & Biostatistics,  The University of Birmingham  B15 2TT | **E:**  **Tel:** | **r.j.lilford@bham.ac.uk**  **0121 414 6772** |
| **Karla Hemming** |  | E:  Tel: | k.hemming@bham.ac.uk  **0121 414 2848** |
| **Peter Chilton** |  | E:  Tel: | p.j.chilton@bham.ac.uk  **0121 414 8288** |

1. By maximum and minimum plausible changes we mean an effect size roughly at the extreme 2·5% and 97·5% limits (i.e. bands on your 95% confidence intervals) [↑](#footnote-ref-1)
2. 1 By maximum and minimum plausible changes we mean an effect size roughly at the extreme 2·5% and 97·5% limits (i.e. bands on your 95% confidence intervals) [↑](#footnote-ref-2)
3. 1 By maximum and minimum plausible changes we mean an effect size roughly at the extreme 2·5% and 97·5% limits (i.e. bands on your 95% confidence intervals) [↑](#footnote-ref-3)
